# PDGFBB improved the biological function of menstrual blood-derived stromal cells and the anti-fibrotic properties of exosomes

**DOI:** 10.1186/s13287-023-03339-y

**Published:** 2023-04-28

**Authors:** Xudong Zhang, Siwen Zhang, Jiarui Qi, Fujie Zhao, Yimeng Lu, Shuyu Li, Shanshan Wu, Pingping Li, Jichun Tan

**Affiliations:** 1 Center of Reproductive Medicine, Department of Obstetrics and Gynecology, Shengjing Hospital of China Medical University, No. 39 Huaxiang Road, Tiexi District, Shenyang, 110022 China; 2 Key Laboratory of Reproductive Dysfunction Disease and Fertility Remodeling of Liaoning Province, No. 39 Huaxiang Road, Tiexi District, Shenyang, 110022 China; 3 Obstetrics and Gynecology Department, Shengjing Hospital of China Medical University, No. 36 Sanhao Street, Heping District, Shenyang, 110022 China

**Keywords:** MenSCs, PDGFBB, Exosomes, Fibrosis, YAP, Intrauterine adhesion

## Abstract

**Background:**

Intrauterine adhesion (IUA) is a reproductive dysfunction disease characterized by endometrial fibrosis, with limited therapeutic options and poor prognosis. Our previous studies confirmed that menstrual blood-derived stromal cells (MenSCs) effectively attenuated endometrial fibrosis in an animal model of IUA mainly through exosomes. This therapeutic effect can be enhanced by platelet-rich plasma (PRP), in which PDGFBB is an abundant growth factor. Therefore, we aimed to compare the effects of PRP and PDGFBB on the biological activities of MenSCs in vitro, and to further investigate the molecular mechanism of MenSCs-derived exosomes in alleviating endometrial fibrosis.

**Methods:**

MenSCs were isolated for in vitro functional assays to examine the viability, migration, and stemness of MenSCs. Endometrial stromal cells (EndoSCs) were treated with 50 ug/ml of MenSCs-derived exosomes, obtained by differential ultracentrifugation extraction. The molecular mechanisms by which PDGFBB improves MenSCs and exosomes alleviate EndoSCs fibrosis were then explored using immunofluorescence, western blot, and co-immunoprecipitation.

**Results:**

Both 100 ng/ml PDGFBB and 10% activated PRP promoted the proliferation, increased the S phase of cell cycle, and inhibited apoptosis of MenSCs in vitro. Compared with PRP, PDGFBB significantly promoted MenSCs migration. All of these effects were inhibited by sorafenib, a PDGFR-β inhibitor. PRP and PDGFBB activated AKT/NF-κB signaling pathway in MenSCs and increased the expression of P65 and OCT4. Moreover, pretreatment of PDGFBB did not increase the secretion of MenSCs but significantly increased the anti-fibrosis effects of MenSCs-derived exosomes on IUA-EndoSCs. MenSCs-derived exosomes attenuated SMAD3 phosphorylation and increased YAP ubiquitination, which reduced the binding of YAP/SMAD3. Pretreatment with PDGFBB amplified this effect.

**Conclusions:**

In summary, PDGFBB could improve the biological functions of MenSCs via AKT/NF-κB signaling pathway, including viability, migration, and stemness. Our results indicated that PDGFBB amplified MenSCs-derived exosomes to attenuate endometrial fibrosis by inhibiting YAP activity, revealing a novel mechanism by which PRP enhanced the ability of MenSCs to repair tissue injury and providing a potential option for improving stem cell efficacy in IUA.

**Supplementary Information:**

The online version contains supplementary material available at 10.1186/s13287-023-03339-y.

## Introduction

Intrauterine adhesion (IUA) is a reproductive dysfunction disease that seriously threatens women's physical and mental health, accounting for 2.8%-45.5% of secondary infertility. The incidence of IUA is increasing annually due to the increased rate of intrauterine procedures such as induced abortion [1]. The pathology of IUA is characterized by the replacement of the endometrial functional layer by a large amount of fiber, and in severe cases, fibrous or myxoid collagen bundles at the lesions may form trans-cavernous fibrous bands, resulting in the reduction or even loss of the uterine cavity volume [2]. Endometrial fibrosis is frequently accompanied by decreased subendometrial blood flow, contributing to difficulties in embryo implantation and miscarriage [3]. Transcervical resection of adhesion is the preferred treatment, but the recurrence rates of patients with severe IUA are as high as 62.5%. Furthermore, only 25% of patients with severe IUA could conceive spontaneously and be at increased risk of pregnancy complications [4, 5].

The Hippo signaling pathway, composed of a series of conserved kinases, is a pathway that primarily inhibits cell growth [6]. Yes-associated protein (YAP) is a key effector molecule downstream of the Hippo pathway and is involved in biological processes such as regulating tissue and organ regeneration, maintaining the balance of cell proliferation and apoptosis, and modulating cell contact inhibition in mammals [7]. Overactivation of YAP has been shown to play an important role in the pathological process of cardiac fibrosis, pulmonary fibrosis, liver fibrosis, renal fibrosis, and skin fibrosis [8]. The level of YAP phosphorylation is decreased in response to inhibition of the Hippo pathway, thus allowing YAP to enter the nucleus and bind to transcription factors (e.g. TEADs) to promote the expression of target genes to regulate cell proliferation and epithelial-mesenchymal transition [9]. Connective tissue growth factor (CTGF) and transforming growth factor (TGF)-β are involved in regulating the degree of tissue remodeling and are among the transcriptional targets of YAP [10]. The results of clinical samples showed that CTGF and TGF-β were highly expressed in the IUA endometrium [11]. Therefore, modulation of YAP activity might be a key target for the prevention and treatment of endometrial fibrosis.

With the ability to self-renew, high proliferation, and multidirectional differentiation, mesenchymal stem cells (MSCs) are considered a promising therapeutic alternative to address the fertility needs of IUA patients. In IUA model animals or clinical studies, bone marrow, umbilical cord, adipose, and endometrium-derived MSCs have been shown to restore uterine cavity morphology, normal menstrual cycle, and fertility [12]. Our team previously extracted mesenchymal stromal cells from menstrual blood (MenSCs) and confirmed through phase I clinical trial that they can significantly increase the thickness and blood vessel density of endometrial, improve endometrial receptivity, and enable successful pregnancy in IUA patients [13]. Further study demonstrated that small extracellular vesicles derived from MenSCs could provide similar therapeutic effects to MenSCs with less migration to other organs [14].

Our previous studies have demonstrated that platelet-rich plasma (PRP) enhanced the biological function of MenSCs and that MenSCs co-transplanted with PRP strengthened the therapeutic effect in the IUA rat model [15, 16]. PRP refers to blood derivatives that have a concentration of platelets exceeding that of physiological platelets, usually derived from autologous whole blood, and is mostly used for tissue repair and regeneration. Platelet activation could release a variety of chemokines and growth factors such as platelet-derived growth factors (PDGFs), TGFs, insulin-like growth factors (IGFs), vascular endothelial growth factor (VEGF), epidermal growth factor (EGF), and fibroblast growth factors (FGFs), which can not only initiate cell migration and colonization to damaged tissues, but also promote cell proliferation, differentiation, and angiogenesis [17, 18]. PDGFs are a major class of growth factors in PRP. PDGF-BB is the most active isoform of the PDGF family, which can bind to all receptors (PDGFRαα, PDGFRαβ, and PDGFRββ) and subsequently activate multiple signaling pathways, facilitating cell migration, survival, and development, and participating in wound repair, tendon remodeling, and ulcer healing [19–21]. Moreover, PDGF-BB has chemotactic effects on neutrophils, macrophages, and other inflammatory cells [19]. An in vitro study revealed that the effect of PDGFBB was similar to the pro-proliferative effect of PRP on adipose-derived MSCs, suggesting that PDGF-BB might be the main effective component of PRP to modify the biological function of MSCs [22].

Therefore, in this study, we aimed to explore the modulatory effect of PDGFBB on the biological function of MenSCs. On this basis, we further investigated the anti-fibrotic effect of exosomes derived from PDGFBB-pretreated MenSCs (EXO^PDGFBB^) on IUA endometrial stromal cells (EndoSCs) in vitro. In addition, we investigated the alterations of the key molecule downstream of the Hippo pathway after the treatment of EXO^PDGFBB^.

## Methods

### MenSCs and EndoSCs isolation and culture

Samples donors were diagnosed with IUA, aged 25–35 years old. Menstrual blood and endometrial samples were obtained from each volunteer. MenSCs were extracted and cultured as described in our previous report [13]. Briefly, menstrual blood samples were obtained on the second day of menses, then transferred to Ficoll (Sigma-Aldrich, St. Louis, MO, USA) and fractionated under density gradient centrifugation according to the manufacturer's instructions. The central cell layer was washed with phosphate-buffered saline (PBS), then seeded in 25-cm^2^ tissue culture bottles (NEST). Cells were cultured in Dulbecco’s modified Eagle medium: nutrient mixture F-12 (Ham’s) (DMEMF12 1:1, HyClone, Logan, UT, USA) supplemented with 10% FBS (Gibco, Waltham, MA, USA) and 1% penicillin–streptomycin (PS; Sigma-Aldrich) at 37 °C with 5% CO_2_. The fresh complete culture medium was replaced after 24 h, and the medium was changed every three days thereafter. After the fusion rate reached 80–90%, the cells were passaged by 0.25% trypsin (Sigma-Aldrich) for further experiments.

The endometrial samples were acquired from volunteers undergoing hysteroscopic transcervical resection of adhesions in the Shengjing Hospital affiliated of China Medical University. The isolation of EndoSCs was modified based on a previously described method [23]. The tissue was cut into pieces less than 1 mm^3^ and digested with 1 mg/ml type I collagenase at 37 °C for 30 min with continuous shaking. The digestion was stopped by adding DMEM/F-12 medium containing 5% FBS and then filtered through a 40 μm filter (CSS013040, Biofil, Guangzhou, China). The filtrate was centrifuged at 300 × *g* for 5 min to acquire EndoSCs, and the subsequent culture method was the same as the above-mentioned MenSCs.

### Platelet-rich plasma (PRP) preparation

Platelet-rich plasma was acquired from the blood transfusion department of Shengjing Hospital and processed as described in our previous study [16]. Thrombin from bovine plasma (T8020, Solarbio, Beijing, China) in 20% CaCl_2_ (Sigma-Aldrich) was added to PRP and incubated at 37 °C for 30 min and 4 °C for 12 h. Only the supernatant was filtered through 0.22-μm sterile filters (Millipore, Carrigwohill, County Cork, Ireland), and then aliquoted and stored at − 80 °C.

### Exosome isolation and identification

The isolation of exosomes referred to our previous study [14]. When MenSCs reached 70% confluence, they were treated with PDGFBB for 24 h. Cells were then washed twice with PBS and cultured in serum-free medium for an additional 24 h. The medium was collected and centrifuged at 2000 × *g* for 10 min to remove dead cells and cell debris. After filtration with a 0.22-μm sterile filter, the supernatant was centrifuged at 10,000 × *g* for 1 h and 100,000 × *g* for 4 h to isolate exosomes. Transmission electron microscopy (TEM) was used to observe the microstructure of exosomes. Nanoparticle tracking analysis (NTA) was used to describe the size distribution and concentration of exosomes. We measured specific surface markers including TSG101 and CD63 using western blot. The exosomal protein concentration was detected by a bicinchoninic acid protein assay (BCA; Epizyme, Shanghai, China), and the absorbance was read at 562 nm with a microplate reader (Multiskan FC, ThermoFisher).

### Cell viability assay

MenSCs viability was tested using the cell counting kit-8 (CCK8; K1018, Apexbio, Houston, Texas, USA) according to the manufacturer's instructions. P3 MenSCs were seeded in 96-well plates at a density of 2000 cells per well for 24 h. Culture media with different concentration gradients of PDGFBB (25, 50, 100, and 200 ng/ml) and 10% PRP were incubated to MenSCs for 7 days. Culture media with 10% FBS was set as the control. Every other day at the same time, 100 μl DMEM/F-12 and 10 μl CCK8 were added and incubated at 37 °C for 3 h. After that, absorbance was measured at 450 nm.

### Flow cytometry

P3 MenSCs were maintained in 100mm^2^ Petri dish (WHB scientific, Shanghai, China) for flow cytometry. The cells were incubated in serum-free medium overnight and then were treated with 10% PRP or PDGFBB for 24 h. The cells were then collected and assayed according to instructions.

For surface marker detection, 1 × 10^5^ cells were resuspended in 200ul of 1% BSA-PBS and incubated with the antibodies of CD34-APC, CD38-PE, CD44-PE, CD45-FITC, CD73-APC, CD90-FITC, CD105-CY5.5 (BD Biosciences, USA). We performed the apoptosis analysis using the Annexin V-FITC/PI apoptosis detection kit (A211-01, Vazyme, Nanjing, China) according to the manufacturer's instructions. Briefly, 5 × 10^5^ cells were collected and washed twice with pre-cooled PBS. Cells were resuspended by 100 μl 1 × binding buffer and incubated with 5 μl of Annexin V-FITC and 5 μl of PI staining solution for 10 min in the dark. Then added 400 μl 1 × binding buffer and analyzed by flow cytometry. For cell cycle analysis, cell cycle analysis kit (FXP0211, 4A Biotech, Beijing, China) was used following the manufacturer’s protocol. Simply, cells were fixed in 95% ethanol-PBS at 4 °C for 24 h, followed by the addition of 400 μl staining buffer, 5 μl PI, and 4 μl RNaseA (2.5 mg/ml) to each tube of cells at 37 °C for 30 min. These cells were analyzed by fluorescence-activated cell sorting using a flow cytometer (BDFACSCalibur; BD Biosciences). Apoptotic rates and phenotypes were analyzed by CytoFLEX (Beckman Coulter, USA), and the cell cycle was quantified by KALUZA 2.2 (Beckman Coulter, USA).

### Wound healing assay and transwell assay

P4 MenSCs were seeded into 6-well plates at a density of 5 × 10^5^ per well and cultured until the cells filled the bottom of the wells. 200 μl Pipette tips were then used to create scratches of the same width in each well. Meanwhile, the medium was replaced with PRP or PDGFBB-containing medium for 24 h. 5 × 10^5^ P4 MenSCs were seeded in the upper layer of transwell chamber (8 μm, ThermoFisher) in 6-well plates with 5% FBS DMEMF12 media or 5% FBS-PDGFBB (100 ng/ml) DMEMF12 media. 10% FBS DMEMF12 media or 10% FBS-PDGFBB (100 ng/ml) DMEMF12 media was added in the lower layer. After culturing for 6 h, the upper layer was stained with 4% PFA and 1% crystal violet. Images were observed at × 100 magnification using an optical microscope (BX53, Olympus, Japan) at 0 and 24 h.

### Immunofluorescence

P3 MenSCs or P4 EndoSCs were cultured on coverslips (14 mm, NEST) at 1 × 10^5^ cells in 6-well plates. After overnight starvation, the medium was replaced with medium containing 100 ng/ml PDGFBB for MenSCs and 50ug/ml EXO or EXO^PDGFBB^ for EndoSCs and incubated for 24 h. For the identification of IUA-EndoSCs, no particular treatment was required. The coverslips were fixed with 4% PFA and then blocked with goat serum (ZLI-9022, ZSGB-Bio, Beijing, China) for 1 h at room temperature. Purified P65 antibody (1:200, #8242, CST), phospho-P65(Ser536) antibody (1:200, #3033, CST), Vimentin antibody (1:300, 10366-1-AP, Proteintech, Chicago, IL, USA), YAP antibody (1:50, 66900-1-Ig, Proteintech), CTGF antibody (1:100, D8Z8U, CST) and phospho-SMAD3 (Ser425) antibody (1:50, YP0585, Immunoway) diluted in PBS were immunostained overnight at 4 °C. The next day, all coverslips were incubated with the secondary antibodies (1:500, Cy3-labeled goat anti-mouse IgG, FITC-labeled goat anti-rabbit IgG, Cy3-labeled goat anti-rabbit IgG, Beyotime, Beijing, China) for 2 h in the dark, and then the nuclei were stained with DAPI (1:20, Beyotime). A fluorescence microscope (Olympus IX73) was employed to observe and CellSens standard software was used to capture images. All images are measured at 1600 × 1200 resolution when acquired. Fluorescence intensity was detected using ImageJ software (National Institutes of Health, Bethesda, MD, USA).

### Western blot

Western blot (WB) was performed as previously described [14, 16]. In brief, cells were washed twice with cold PBS and then lysed with fresh RIPA lysis buffer (PC101, Vazyme) containing protease inhibitor cocktail (GRF101, Vazyme) and phosphatase inhibitor cocktail (GRF102, Vazyme). Total protein concentration was determined using the BCA method. Equal amounts of protein from each sample were separated by 10% sodium dodecyl sulphate (SDS) polyacrylamide gels and transferred to 0.45 μm PVDF membranes (EMD Millipore, Billerica, MA, USA). Afterward, the membranes were incubated overnight at 4℃ with primary antibodies including AKT (#4691, CST, 1:1000), phospho-AKT (Thr308) (#13038, CST, 1:1000), IKKα (#2682, CST, 1:1000), IKKβ (#2684, CST, 1:1000), IκB (#4812, CST, 1:1000), phospho-IκB (Ser32) (#2859, CST, 1:1000), phospho-IKKα/β (Ser176/180) (#2697, CST, 1:1000), NF-κB p65 (#8242, CST, 1:1000), Collagen I (14695-1-AP, proteintech, 1:2000), CTGF (WL02602, Wanleibio, Shenyang, China, 1:500), YAP (13584-1-AP, proteintech, 1:2000), phospho-YAP (Ser127) (#13008, CST, 1:1000), SMAD3 (#9513, CST, 1:1000), phospho-SMAD3 (Ser425) (YP0585, Immunoway, 1:1000), Ubiquitin (ab134953, Abcam, Cambridge, MA, USA, 1:1000), β-Tubulin (M20005S, Abmart, 1:2000) and GAPDH (#5174, CST, 1:1000). The membranes were then incubated with the appropriate secondary antibodies for 2 h at room temperature. The signals were visualized with ECL (#1862420, #1862421, Thermo Fisher Scientific, Waltham, MA, USA), and the density of protein bands was semi-quantified using ImageJ.

### Co-immunoprecipitation assay

EndoSCs were harvested by the immunoprecipitation lysis buffer. The proteins were immunoprecipitated by incubating with YAP in incubation buffer at 4 °C overnight. Afterward, pre-cleared protein A-sepharose beads were added and incubated at 4 °C for 4 h. The precipitated complexes were then eluted with the elution buffer. The eluted samples were then subjected to western blot as described above.

### Statistical analysis

Descriptive statistics and statistical analysis were carried out using GraphPad Prism 8 (San Diego, CA, USA). Quantitative data are presented as the means ± SD. Multiple group comparisons were performed using one-way ANOVA, and Student’s *t*-test was applied to analyze two-group comparisons. A value of *P* < 0.05 was considered statistically significant (*P < 0.05, **P < 0.01, ***P < 0.001).

## Results

### PDGFBB improved the viability while inhibiting the apoptosis of MenSCs in vitro

**Fig. 1:**
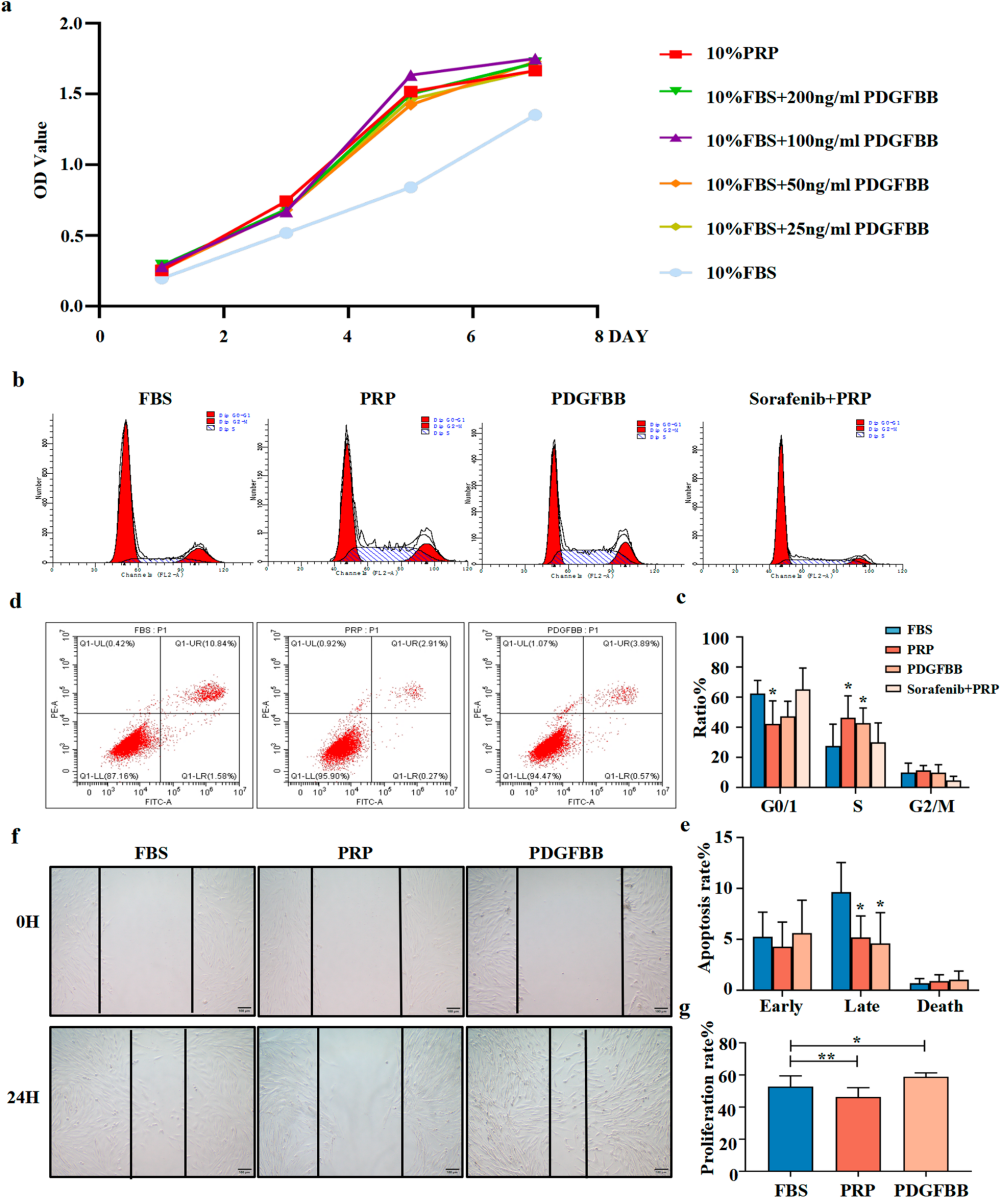
PDGFBB promoted MenSCs viability and inhibited apoptosis. **a** CCK8 assay detected the viability of P3 MenSCs cultured with different concentrations of PDGFBB, 10%PRP or 10% FBS; **b** Flow cytometry analysis of cell cycle of P3 MenSCs after treatment for 24 h; **c** Cell cycle statistics histogram; **d** Flow cytometry detection of cell apoptosis by Annexin V/PI staining. P3 MenSCs were treated for 24 h and then were tested; **e** Early apoptosis, late apoptosis, and death rate of P3 MenSCs after treatment for 24 h; **f** Wound healing assay of P3 MenSCs after treatment for 24 h; **g** Cell migration statistics histogram. Data were mean ± SD, *P < 0.05, **P < 0.01, ***P < 0.001 for One Way ANOVA

First, the cell counting kit-8 (CCK8) assay was used to examine the effect of PDGFBB on the growth of IUA-MenSCs. As shown in Fig. 1a, compared with the control group, PDGFBB at various concentrations improved the viability of IUA-MenSCs. The optical density (OD) value of each PDGFBB-supplemented group was significantly higher than that of the FBS group from day 3 and lasted until day 7. The trend of the viability curve in each PDGFBB-supplemented group was similar to that of the 10% PRP group. 100 ng/ml PDGFBB supplement showed the best effect of improving the viability of MenSCs. Hence, all further experiments were conducted with 10% FBS + 100 ng/ml PDGFBB.

Subsequently, we performed flow cytometry to detect the cell cycle in the different treatment groups. As shown in Fig. 1b, c, after 10% PRP or 100 ng/ml PDGFBB treatment for 24 h, the proportion of cells in the S phase was significantly increased compared with that in the FBS group (*P*_*PRP*_ = 0.047, *P*_*PDGFBB*_ = 0.049, respectively). However, Sorafenib, a PDGFR-β receptor inhibitor, was able to inhibit the PRP-induced increase in the number of cells in the S phase. Compared with the FBS group, the proportion of cells in the G0/G1 phase in the PRP group was significantly decreased, and there were no differences between groups for cells in the G2/M phase. Moreover, as shown in Fig. 1d, e, the late apoptosis (Annexin V^+^/PI^+^) of MenSCs was significantly inhibited after PRP or PDGFBB treatment for 24 h, compared to the FBS group (*P*_*PRP*_ = 0.028, *P*_*PDGFBB*_ = 0.022, respectively). Meanwhile, there was no significant difference in apoptosis between the PRP group and PDGFBB group. These data suggested that PDGFBB acted similarly to PRP, enhancing the proliferation of MenSCs by promoting DNA replication and inhibiting apoptosis.

To further explore the role of PDGFBB, a wound-healing assay was utilized to measure the migration ability of IUA-MenSCs. As shown in Fig. 1f, g, compared with the FBS group, the PDGFBB group showed a significant narrower scratch area and higher wound closure rate after 24 h, while the PRP group showed a significantly lower wound closure rate (*P*_*PRP*_ = 0.002, *P*_*PDGFBB*_ = 0.001, respectively). The results of the transwell assay were consistent with the wound-healing assay, in which more MenSCs were shuttled to the lower compartment in the PDGFBB group (Additional file 1: Fig. S1d). This result suggested that PDGFBB could promote the migration of IUA-MenSCs cultured in vitro, while PRP had an inhibitory effect on the migration of IUA-MenSCs. This indicated that PDGFBB might be a crucial growth factor in PRP that promoted the proliferation of IUA-MenSCs.

### PDGFBB promoted the expression of stemness marker OCT4

**Fig. 2:**
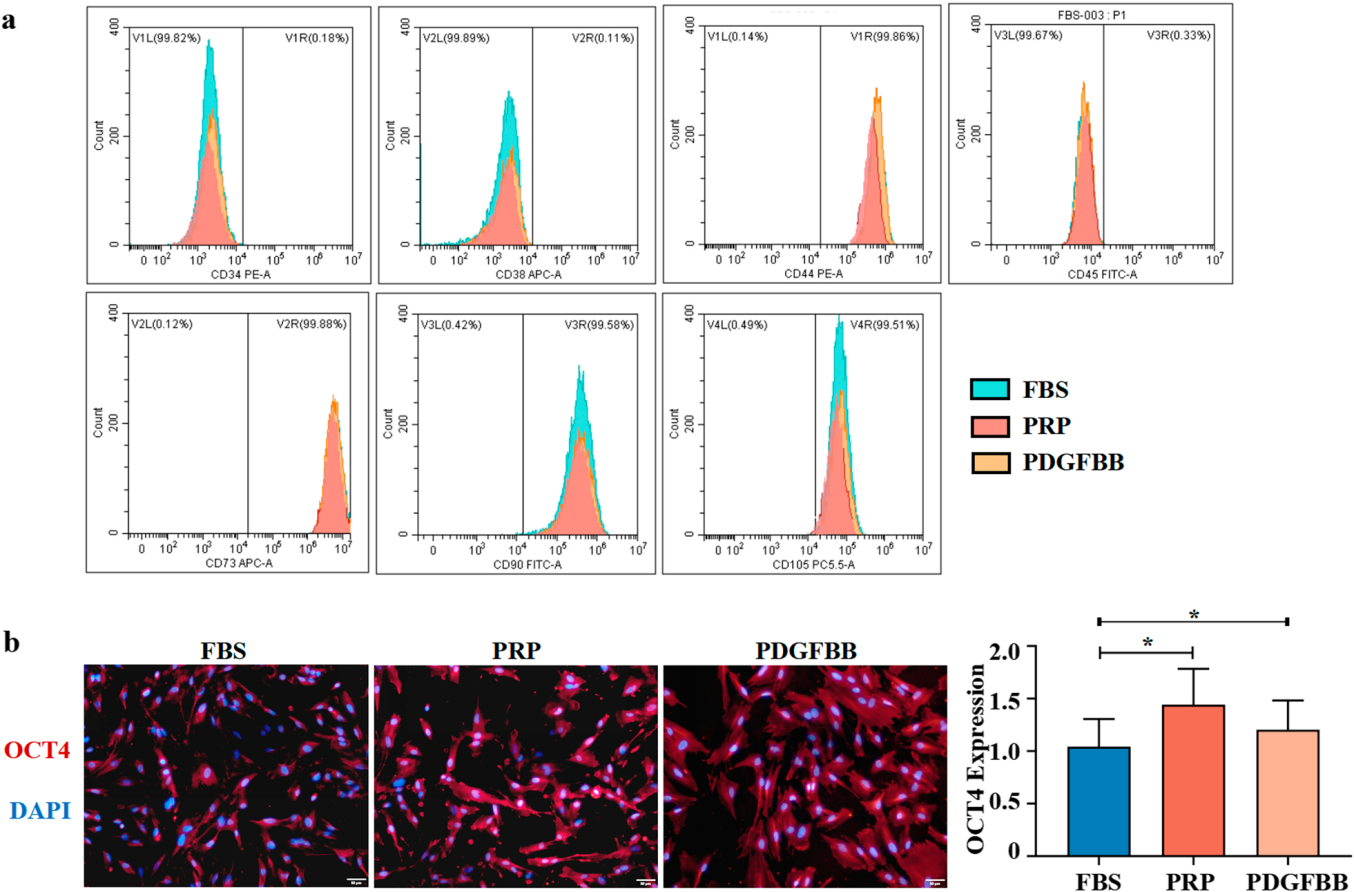
PDGFBB sustained the expression of mesenchymal stem cell markers and promoted the expression of OCT4. **a** Flow cytometry analysis of MSCs surface markers CD34, CD38, CD44, CD45, CD73, CD90, and CD105. **b** Immunofluorescence assays of OCT4 (Red) and DAPI was used to stain nuclei (Blue). Fluorescence intensity analysis of OCT4. Data were mean ± SD, *P < 0.05, **P < 0.01, ***P < 0.001 for One Way ANOVA

Flow cytometry was applied to evaluate changes in stemness markers of MenSCs following treatment with PRP and PDGFBB. As shown in Fig. 2a, there were no significant differences in the expression of CD34, CD38, CD44, CD45, CD73, CD90, and CD105 after treatment for 24 h. Immunofluorescence showed that there was increased expression of OCT4 in MenSCs in the PRP group and PDGFBB group. As shown in Fig. 2b, the intensities of OCT4, a marker of pluripotency and stemness were significantly increased in the PRP group and PDGFBB group (*P*_*PRP*_ 0.049, *P*_*PDGFBB*_ = 0.024, respectively).

### Pretreatment with PDGFBB didn’t affect the paracrine of MenSCs in vitro

**Fig. 3:**
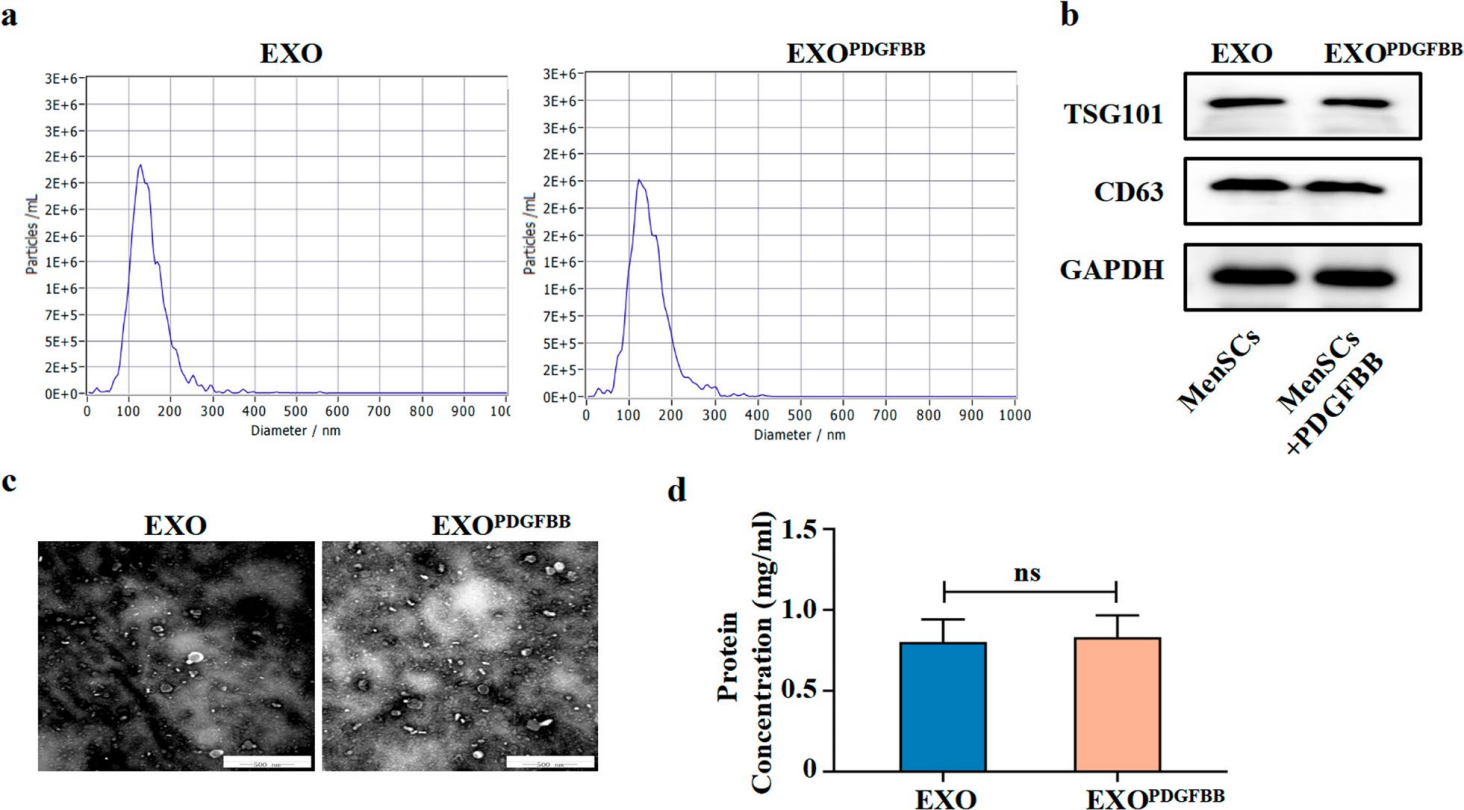
PDGFBB pretreatment did not affect the secretory properties of MenSCs. **a** Size distribution of MenSCs-EXO examined by NTA; **b** Western blot of CD63 and TSG101 of MenSCs-EXO; Full-length blots are presented in Additional file 2: Fig. S2a; **c** Morphology of MenSCs-EXO observed by TEM (scale bar = 500 nm); **d** BCA analysis of EXO protein concentration. Data were mean ± SD, *P < 0.05, **P < 0.01, ***P < 0.001 for One Way ANOVA

We determined whether PDGFBB treatment could influence the paracrine activity of IUA-MenSCs in vitro. MenSCs were treated with or without PDGFBB for 24 h and exosomes were isolated from serum-free media after 24 h of incubation. TEM, NTA, western blot, and BCA were used for the identification and analysis of exosomes. As shown in Fig. 3a, c, TEM revealed typically nanoparticles and NTA showed a similar size distribution (average 128.0 nm versus 128.3 nm) in both groups. The protein levels of exosome surface markers including CD63 and TSG101 did not differ with PDGFBB treatment (Fig. 3b) (Additional file 2: Fig. S2a). Furthermore, there was no difference in the protein concentration of exosomes between the two groups (Fig. 3d). The above results indicated that PDGFBB did not affect the size, shape, electron density, and protein content of exosomes secreted by MenSCs.

### PDGFBB activated AKT and nuclear factor-κ-gene binding (NF-κB) pathways in IUA-MenSCs

**Fig. 4:**
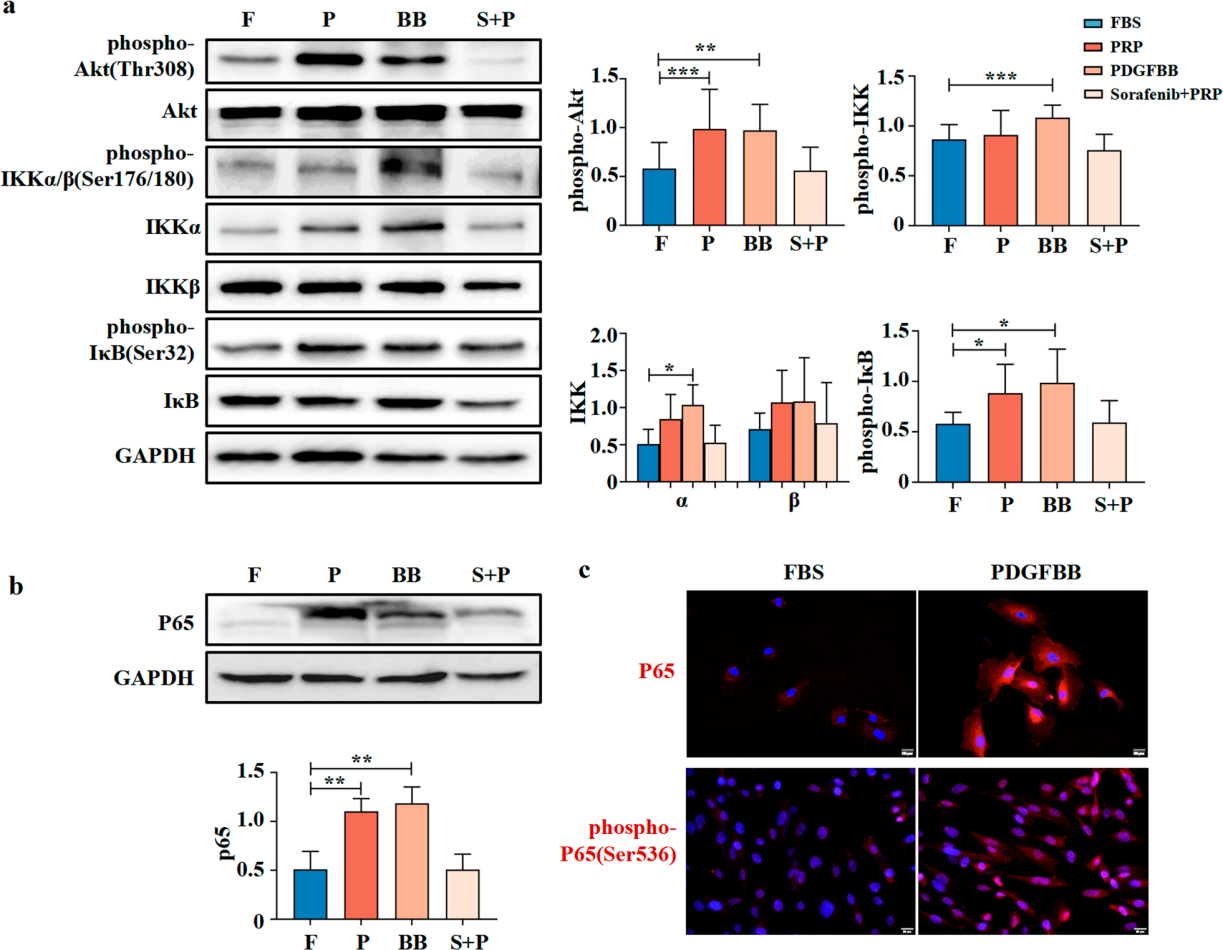
PDGFBB activated the AKT/ NF-κB signaling pathway among groups. **a** Western blot of molecules of Akt/NF-κB signaling pathway among FBS group, PRP group, PDGFBB group, and sorafenib group; Full-length blots are presented in Additional file 2: Fig. S2b; **b** Western blot of P65 among FBS group, PRP group, PDGFBB group, and sorafenib group; Full-length blots are presented in Additional file 2: Fig. S2d; **c** Immunofluorescence assays of P65 and phospho-P65 (Red), and DAPI was used to stain nuclei (Blue). Data were mean ± SD, *P < 0.05, **P < 0.01, ***P < 0.001 for One Way ANOVA. F: FBS group; P: PRP group; BB: PDGFBB group; S + P: Sorafenib and PRP group

Recent studies have revealed that the AKT signaling pathway might influence the properties of stem cells and play a vital role in maintaining their growth, survival, proliferation, and differentiation [24]. NF-κB signaling, which could be activated by AKT, holds an important regulatory role in tissue damage and repair [25, 26]. We then analyzed the expression levels of AKT and NF-κB pathway-related proteins in IUA-MenSCs after treatment with PRP, PDGFBB, or PRP and sorafenib for 24 h. As shown in Fig. 4a (Additional file 2: Fig. S2b), the levels of phospho-AKT (Thr308, *P* < 0.01) and phospho-IκB (Ser32, *P* < 0.05) were significantly increased after PRP and PDGFBB treatment. Compared with the FBS group, there was also a significant increase in the expression levels of IKKα (*P* < 0.05) and phospho-IKKα/β (Ser176/180, *P* < 0.05) in the PDGFBB group. Sorafenib inhibited the PRP-mediated increase in phosphor-AKT and phospho-IκB.

Moreover, as shown in Fig. 4b (Additional file 2: Fig. S2d), the level of P65, a key molecule of the NF-κB signaling pathway, was significantly increased in IUA-MenSCs following PRP and PDGFBB treatment for 24 h (*P* < 0.01). Immunofluorescence results showed that PDGFBB promoted the expression and nuclear translocation of P65 and phospho-P65 (Ser536) in IUA-MenSCs (Fig. 4c). All these results suggested that AKT/NF-κB signaling pathway may be a potential molecular pathway for PDGFBB to act on IUA-MenSCs.

### EXO^PDGFBB^ significantly suppressed the expression of Collagen I, CTGF, YAP, and SMAD3 in IUA-EndoSCs

**Fig. 5:**
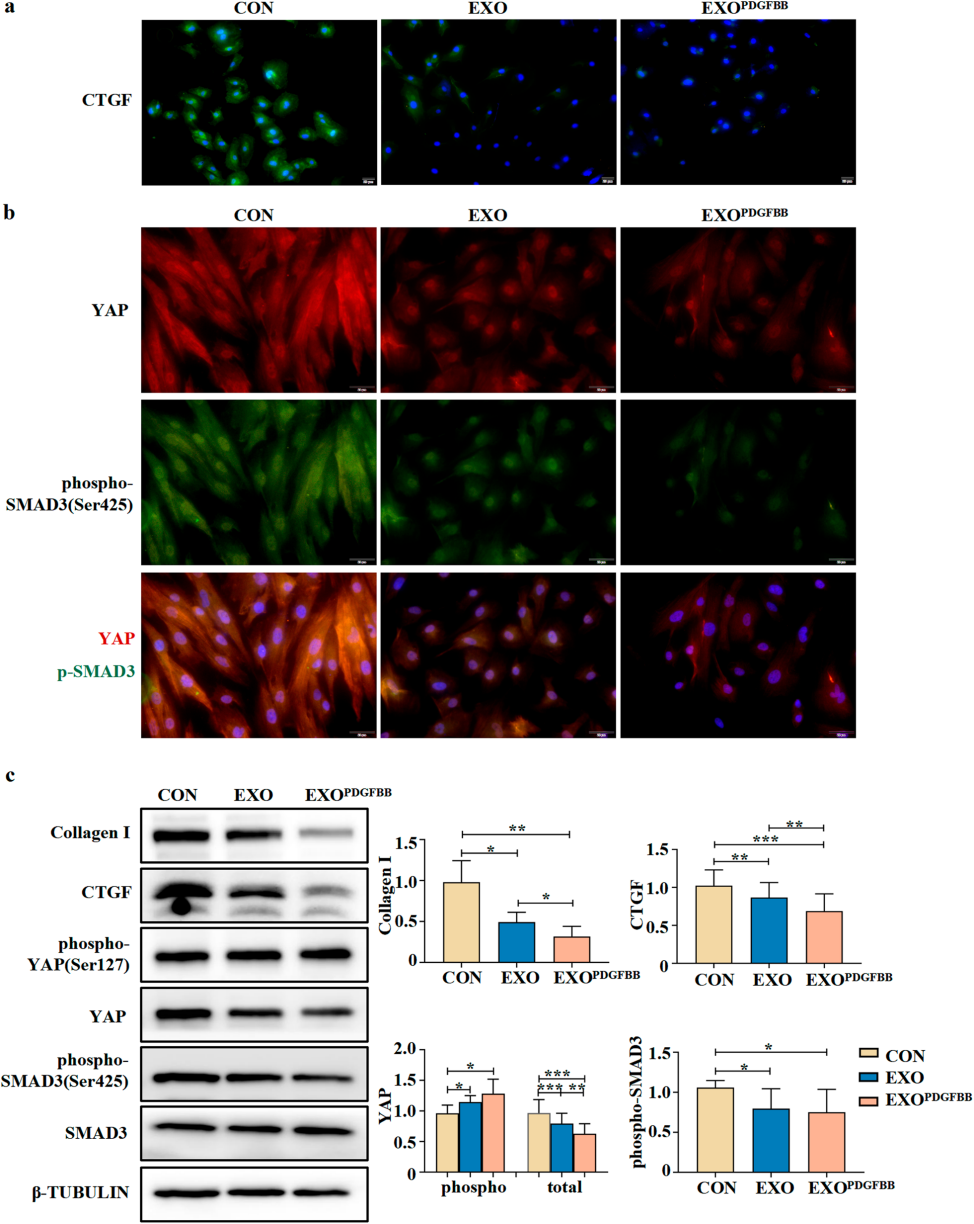
Comparison of protein expression of Collagen I, CTGF, YAP, and SMAD3 after treatment with EXO and EXO^PDGFBB^. **a** Immunofluorescence assays of CTGF (Green). DAPI was used to stain nuclei (Blue). **b** Immunofluorescence assays of YAP (Red) and phospho-SMAD3 (Green). DAPI was used to stain nuclei (Blue). **c** Western blots of molecules expression of Collagen I, CTGF, phospho-YAP, YAP, phospho-SMAD3, and SMAD3 among the control group, EXO group, and EXO^PDGFBB^ group. Full-length blots are presented in Additional file 2: Fig. S2c. Data were mean ± SD, *P < 0.05, **P < 0.01, ***P < 0.001 for One Way ANOVA

To investigate whether EXO^PDGFBB^ could alleviate endometrial fibrosis, we extracted EndoSCs from clinical specimens of IUA patients (IUA-EndoSCs). IUA-EndoSCs are spindle-shaped and positive for vimentin (Additional file 1: Fig. S1b). A higher expression of CTGF and YAP in IUA-EndoSCs was observed in IUA-EndoSCs compared with the normal EndoSCs (N-EndoSCs) (Additional file 1: Fig. S1c). In addition, the expression levels of YAP and fibrosis-related proteins including Collagen I and CTGF in the IUA-EndoSCs were significantly higher than those in N-EndoSCs (Additional file 1: Fig. S1a; Additional file 3: Fig. S3b). We treated IUA-EndoSCs with MenSCs-derived exosomes (EXO) and EXO^PDGFBB^ for 24 h, respectively. As shown in Fig. 5a, b, both EXO and EXO^PDGFBB^ significantly suppressed the expression of CTGF, YAP, and phospho-SMAD3 (Ser425) compared with the CON group. Moreover, the nuclear translocation of YAP and phospho-SMAD3 in IUA-EndoSCs was significantly decreased after EXO and EXO^PDGFBB^ treatment.

According to the results of western blot (Fig. 5c) (Additional file 2: Fig. S2c), the expression of Collagen I, CTGF, YAP, and phospho-SMAD3 (Ser425) in IUA-EndoSCs were reduced after treatment of EXO and EXO^PDGFBB^, respectively (*P* < 0.05). The expression level of YAP was lower in the EXO^PDGFBB^ group (*P* < 0.01). Meanwhile, the expression of phospho-YAP (Ser127) was significantly increased in the EXO group and EXO^PDGFBB^ group (*P* < 0.05).

**Fig. 6:**
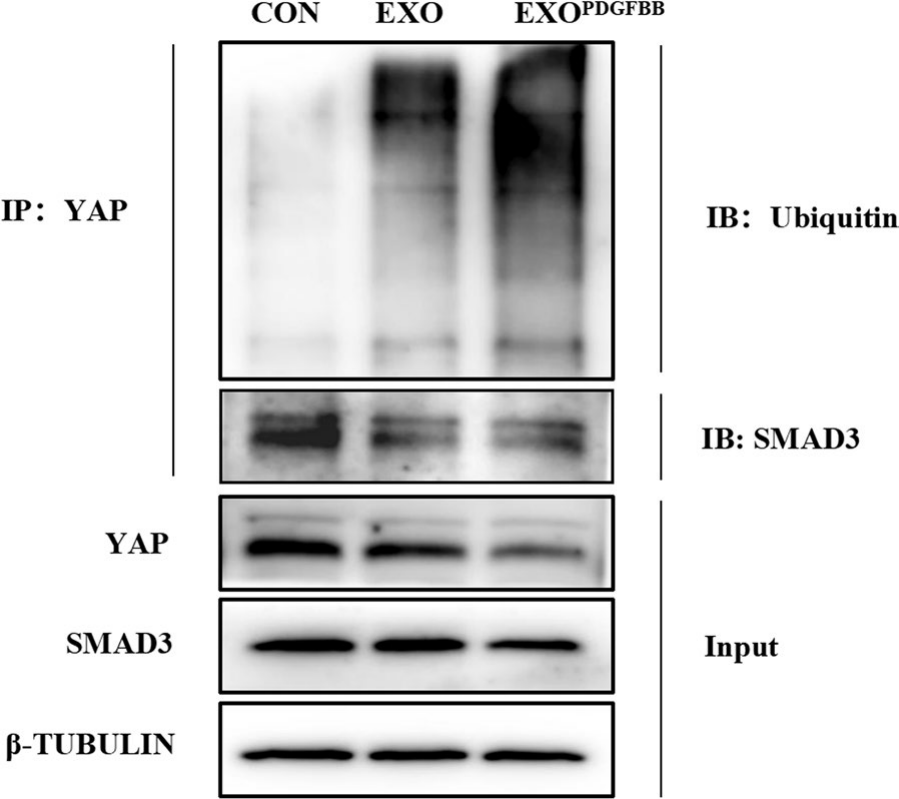
EXO^PDGFBB^ promoted the ubiquitination of YAP and reduced its binding to SMAD3. The ubiquitination of YAP protein with EXO and EXO^PDGFBB^ treatment was determined by co-IP. Full-length blots are presented in Additional file 3: Fig. S3a

**Fig. 7:**
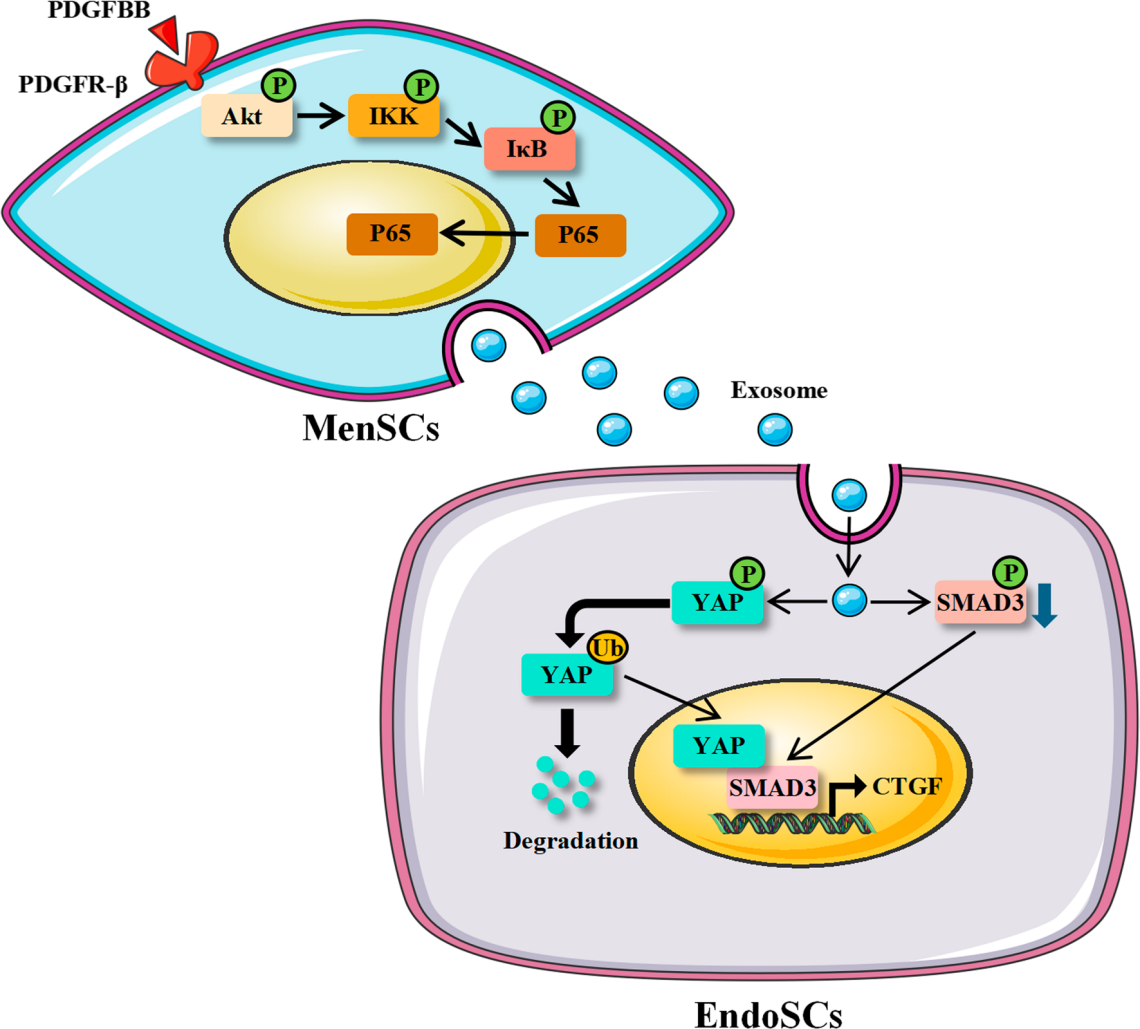
The mechanism graph of the regulatory network. A proposed hypothesis for EXO^PDGFBB^ attenuated EndoSCs fibrosis through YAP ubiquitination and degradation. Figure 7 was modified from Servier Medical Art (http://smart.servier.com/), licensed under a Creative Common Attribution 3.0 Generic License. (https://creativecommons.org/licenses/by/3.0/)

Furthermore, a co-immunoprecipitation (co-IP) assay was conducted to detect the effect of EXO^PDGFBB^ in the alteration of YAP and SMAD3. As shown in Fig. 6 (Additional file 3: Fig. S3a), the ubiquitinated modification of YAP was significantly increased after EXO and EXO^PDGFBB^ treatment in IUA-EndoSCs with the decline of YAP expression. Moreover, the EXO^PDGFBB^ significantly inhibited the binding of YAP and SMAD3. Therefore, the above data indicated that EXO and EXO^PDGFBB^ effectively reduced the fibrosis of IUA-EndoSCs by regulating YAP and SMAD3, and EXO^PDGFBB^ increased the level of YAP ubiquitination and reduced the binding of YAP and SMAD3 (Fig. 7).

## Discussion

This study demonstrated that PDGFBB had similar effects on the biological functions of MenSCs as PRP, including promoting proliferation, inhibiting apoptosis, and improving stemness, but had the opposite effect on migration compared with PRP. Furthermore, PDGFBB pretreatment did not affect the secretion of exosomes from MenSCs, whereas EXO^PDGFBB^ could suppress the expression of fibrosis-related genes by promoting YAP ubiquitination and decreasing its binding to SMAD3, thus alleviating endometrial fibrosis. These results suggested that PDGFBB might be a key growth factor in PRP to enhance the therapeutic effect of MenSCs.

PDGFs promote cell proliferation, survival, and migration, primarily effect on cells of mesenchymal origin [27]. Supplementing the culture media with PDGFBB could support MSCs properties. PDGFBB promoted the proliferation of human periodontal ligament-derived MSCs and increased the expression of stem cell markers such as CD146 and CD105 with a concentration of 50 ng/ml [28]. This is generally consistent with our study, where PDGFBB promoted proliferation and maintained the stemness of MenSCs. The major difference is that the optimal pro-proliferation concentration in the present study was 100 ng/ml, which might be due to the different origins of the cells and the fact that the culture systems were not identical (the medium in this study included FBS). Min et al. [29] proposed that IUA patients had a lower proportion of CD146 + PDGFR + side population of endometrial cells and had functional differences. Moreover, supplementation of PDGFBB in the 3D culture system not only enhanced the proliferation and stemness of MSCs but also stimulated the progressive migration of MSCs through the microcarrier, thus strengthening the tissue repair and regenerative benefits [30, 31]. This study revealed that one of the differences between PDGFBB and PRP was that the former stimulated the migration of MenSCs, while the latter acted as an inhibitor.

AKT, also known as Protein Kinase B (PKB), can phosphorylate numerous downstream proteins, of which IKK is activated and cross-linked with the NF-κB pathway, regulating a range of cellular activities such as growth, survival, proliferation, and glucose metabolism [32]. Our previous in vitro study revealed that PRP could modify the biological function of MenSCs by activating the AKT pathway [16], and a similar conclusion was reported by Ji et al. [33] regarding umbilical cord-derived MSCs. In this study, the results suggested that PDGFBB could also regulate the AKT/NF-κB pathway and promote the nuclear translocation of the downstream key molecule P65, thereby improving the survival and proliferation of MenSCs. According to the study by Wang et al*.* [34], a combined treatment of MenSCs and PDGFBB significantly promoted endometrial epithelial cell proliferation, migration, and angiogenesis, and accelerated intrauterine repair compared to MenSCs only. In tissue repair remodeling, MSCs have a very limited role in proliferation and differentiation into target cells, relying more on their paracrine effect, of which exosomes might be a key mediator. Exosomes are capable of carrying specific bioactive components (proteins, lipids, nucleic acids) similar to those of their originating cells and can be transferred to the site of injury to perform biological functions such as promoting cell proliferation, reversing tissue fibrosis, modulating immune responses, and alleviating inflammatory responses [35]. Our preclinical study demonstrated that MenSCs-derived microvesicles are a safe and effective treatment that could restore the morphology of endometrium, promote glandular regeneration and angiogenesis, attenuate endometrial fibrosis, and significantly improve endometrial receptivity and pregnancy outcomes with multiple transplantations [14]. The present study suggested that PDGFBB did not significantly increase the total amount of protein secreted by MenSCs, hence we hypothesized that PDGFBB treatment could affect the exosome cargos of MenSCs.

YAP is an important transcriptional coactivator that can contribute to tissue remodeling, regeneration, and epithelial metaplasia. It is mainly targeted in the nucleus and binds to transcription factors such as the TEADs, SMADs, RUNX1/2, P73, and ErbB4 to regulate the expression of a range of downstream genes [36]. Piersma et al*.* [37] revealed that YAP levels and nuclear localization were significantly elevated in affected Dupuytren disease tissue, a fibrotic disorder, and that knockdown of YAP reduced α-SMA formation and collagen I deposition. The study of Jin et al*.* [38] demonstrated that the YAP inhibitor verteporfin significantly lowered CTGF and TGF-β levels in the renal tubulointerstitial and alleviated renal fibrosis due to unilateral ureteral obstruction (UUO). Hippo signaling activation might be a key driver to suppress YAP activity. According to the work by Zhu et al*.* [39], MenSCs activated the Hippo/TAZ pathway in EndoSCs inhibiting the nuclear translocation of TAZ mainly through paracrine. Similarly, we observed that the level and nuclear localization of YAP in EndoSCs of IUA patients were also higher than those of normal subjects and that MenSCs-derived exosomes lowered the level of YAP in IUA-EndoSCs, inhibited YAP translocation into the nucleus, and subsequently lowered CTGF transcription level and attenuated fibrosis. Furthermore, the YAP degradation regulated by metabolic enzymes is another access mechanism resulting in YAP inactivation. Ji et al*.* [40] indicated that CK1δ and β-TRCP in human umbilical cord MSCs-derived exosomes mediated ubiquitination modification and degradation of YAP, thereby reducing the intranuclear transfer of YAP and attenuating renal fibrosis due to UUO. We observed that the phosphorylation and ubiquitination of YAP in IUA-EndoSCs were both enhanced after treatment of exosomes derived from MenSCs, suggesting the activation of the Hippo pathway and an enzyme-regulated YAP degradation contributed to the suppression of YAP activity. Moreover, PDGFBB-pretreated MenSCs-derived exosomes were able to further lower the level of YAP, implying a ubiquitin ligases-secreted promotion in MenSCs by PDGFBB.

SMAD3 is a critical transcriptional regulator of the TGF-β signaling, responsible for translocating TGF-β signaling from the cell membrane surface into the nucleus, where it binds to intranuclear-related factors and regulates the expression of target genes. Silencing of SMAD3 inhibited TGF-β-induced expression of CTGF in fibroblasts [41]. Based on a previous in vivo study, we further demonstrated that MenSCs-derived exosomes inhibited the phosphorylation activation of SMAD3, consequently attenuating fibrosis in the IUA EndoSCs. YAP/TAZ has been identified as vital for the functioning of the TGF-β/SMAD signaling pathway [42–45]. In maintaining the multidirectional differentiation and self-renewal potential of embryonic stem cells, the absence of TAZ inhibited TGF-β signaling, and the SMAD2/3–4 complexes failed to accumulate in the nucleus and activate transcription, resulting in the differentiation of embryonic stem cells to neuroectodermal lineages [44]. Likewise, Grannas et al*.* [43] proved that TGF-β induced the formation of the YAP-SMAD2/3 complexes, which were predominantly located in the nucleus and regulated downstream gene expression under sparse cell conditions. Recently, Wang et al*.* [45] demonstrated that the nuclear binding and transcriptional regulation of YAP/SMAD3 is critical for the development of bladder fibrosis. In fibrotic diseases, both the Hippo/YAP and TGF-β/Smad pathways could be regulated by matrix stiffness to impact the fibrotic phenotype, and blocking their cross-linking has been found to be a potential therapeutic target for fibrosis. A study on renal fibrosis suggested that inhibition or knockout of YAP downregulated the expression of SMAD2/3, mainly reducing the nuclear localization of SMAD2/3, thereby effectively alleviating renal fibrosis caused by UUO [42]. Our findings are consistent with the above, that MenSCs-derived exosomes could lower the expression and nuclear localization of YAP and SMAD3 in IUA-EndoSCs, decrease YAP-SMAD3 complex formation, and subsequently inhibit the fibrotic phenotype. This might be precisely due to the lack of YAP blocking the further transmission of TGF-β signaling.

## Conclusion

In conclusion, we demonstrated that PDGFBB acted similarly to PRP to activate the AKT/NF-κB signaling pathway to improve the biological properties of MenSCs in vitro. PDGFBB pretreated MenSCs-derived exosomes alleviate fibrosis in IUA-EndoSCs by promoting YAP phosphorylation modification and ubiquitination degradation to reduce its binding to SMAD3. PDGFBB could enhance the therapeutic effect of MenSCs-derived exosomes, a biogenic nanotherapeutics, providing new insight into anti-fibrotic therapy.

## Supplementary Information

**Additional file 1: Fig. S1.** Comparison of N-EndoSCs and IUA-EndoSCs and the transwell assay of the PDGFBB group.

**Additional file 2: Fig. S2.** Corresponding uncropped full-length gels and blot.

**Additional file 3: Fig. S3.** Corresponding uncropped full-length gels and blot.

## Data Availability

The datasets generated during and/or analyzed during the current study are available from the corresponding author upon reasonable request.

## Abbreviations

CCK8: Cell counting kit-8
CTGF: Connective tissue growth factor
EGF: Epidermal growth factor
EndoSCs: Endometrial stromal cells
FGF: Fibroblast growth factor
IGF: Insulin-like growth factor
IUA: Intrauterine adhesion
MenSCs: Menstrual blood-derived stromal cells
NTA: Nanoparticle tracking analysis
PDGF: Platelet-derived growth factor
PRP: Platelet-rich plasma
TEM: Transmission electron microscopy
TGF: Transforming growth factor
UUO: Unilateral ureteral obstruction
VEGF: Vascular endothelial growth factor
YAP: Yes-associated protein
