# Supplementary figures and images for: PDGFBB improved the biological function of menstrual blood-derived stromal cells and the anti-fibrotic properties of exosomes
Source: Stem Cell Res Ther. 2023 Apr 28;14:113. doi: 10.1186/s13287-023-03339-y (PMC10148410; doi:10.1186/s13287-023-03339-y)

**a**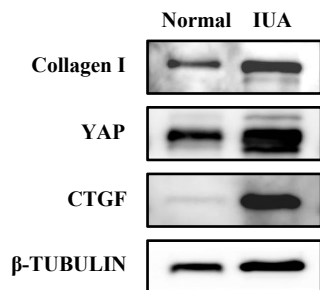**b**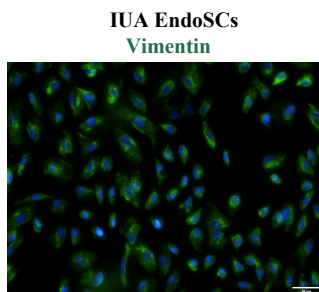**c**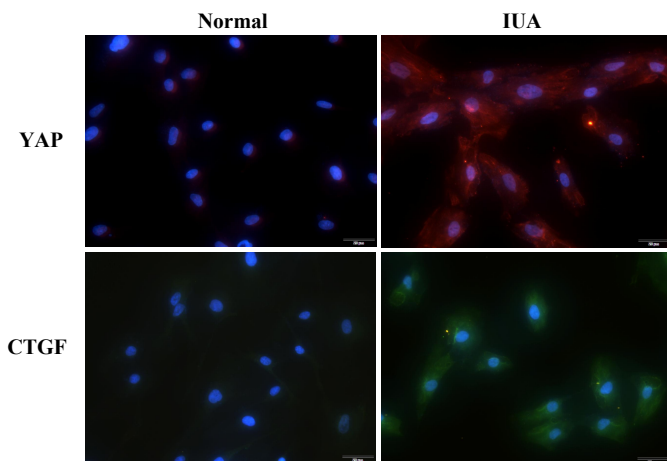**d**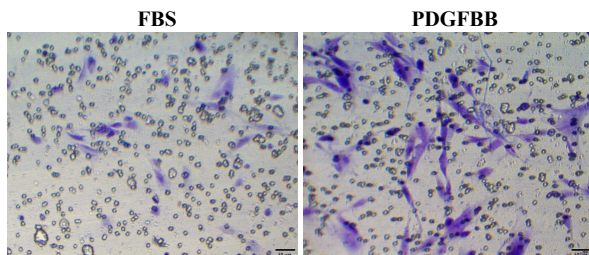

Supplement: Supplementary file 1 — Additional file 1: Fig. S1. Comparison of N-EndoSCs and IUA-EndoSCs and the transwell assay of the PDGFBB group. [file 13287_2023_3339_MOESM1_ESM.pdf]

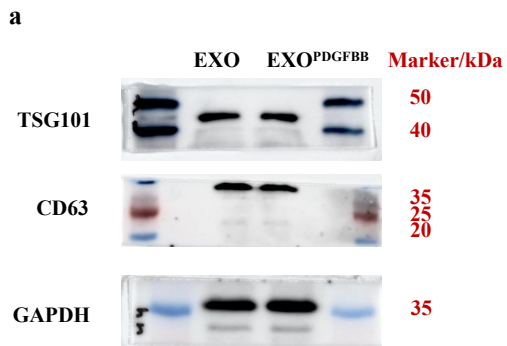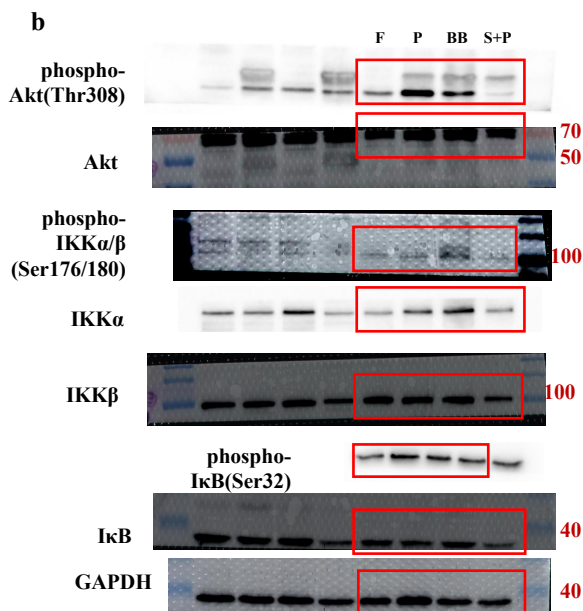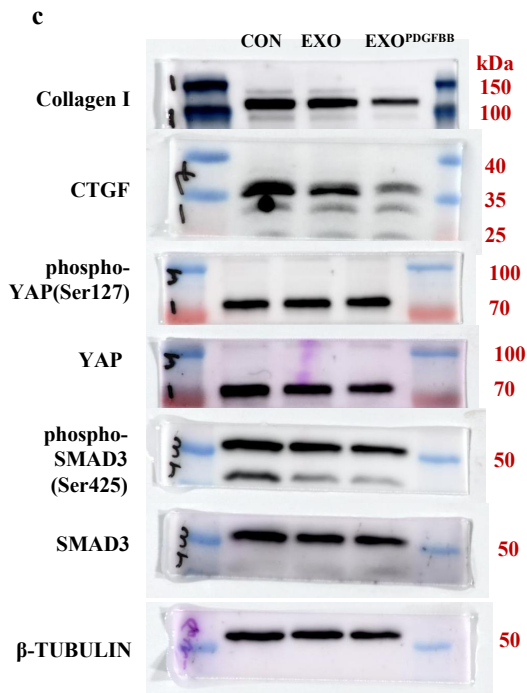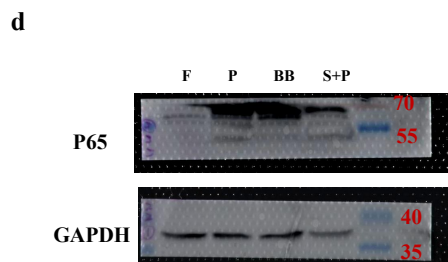

Supplement: Supplementary file 2 — Additional file 2: Fig. S2. Corresponding uncropped full-length gels and blot. [file 13287_2023_3339_MOESM2_ESM.pdf]

**a**

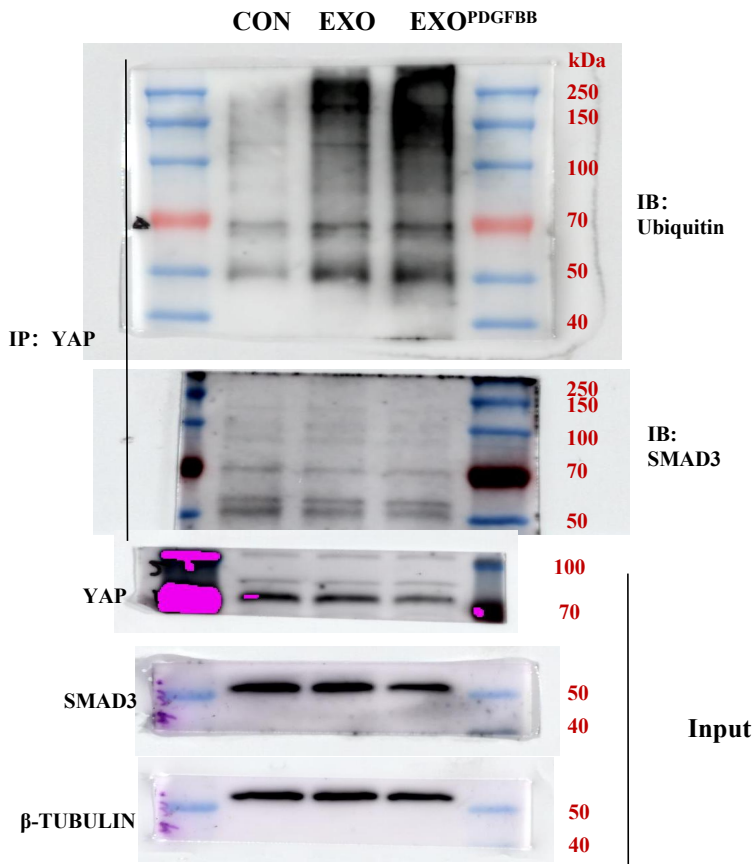

**b**

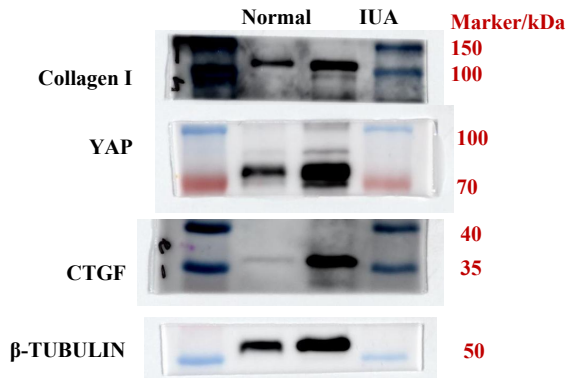

Supplement: Supplementary file 3 — Additional file 3: Fig. S3. Corresponding uncropped full-length gels and blot. [file 13287_2023_3339_MOESM3_ESM.pdf]
